# Costs of health and social services use in children of parents with mental illness

**DOI:** 10.1186/s13034-021-00360-y

**Published:** 2021-02-20

**Authors:** Tamara Waldmann, Maja Stiawa, Ümügülsüm Dinc, Gülsah Saglam, Mareike Busmann, Anne Daubmann, Bonnie Adema, Karl Wegscheider, Silke Wiegand-Grefe, Reinhold Kilian

**Affiliations:** 1 Department of Psychiatry and Psychotherapy II, University of Ulm and BKH Günzburg, Lindenallee 2, 89312 Günzburg, Germany; 2 Department of Child and Adolescent Psychiatry and Psychotherapy, University Medical Center Hamburg-Eppendorf, Martinistraße 52, 20246 Hamburg, Germany; 3 Department of Medical Biometry and Epidemiology, University Medical Center Hamburg-Eppendorf, Martinistraße 52, 20246 Hamburg, Germany

**Keywords:** Costs, Children of parents with mental illness, Health service use, Social service use

## Abstract

**Background:**

Children of parents with mental illness have a higher risk of developing mental health problems when compared with the general population. Therefore, families with parents with mental illness are a suitable target group for selective prevention. In order to plan and evaluate the health economic consequences of preventive interventions for this target group, data on the societal costs related to parenthood under the condition of mental disorders are needed. To date, within Germany there has been a lack of research evaluating the costs of mental health treatment and use of social services by children and adolescents with parents with mental illness.

**Methods:**

As part of a multicentre randomised controlled trial, use and costs of health and social services were assessed for a sample of 332 children and adolescents with parents with mental illness in six regions of Germany. Service use at baseline was assessed by the German version of the Children and Adolescent Mental Health Service Receipt Inventory. Costs were calculated for 12 months based on diagnosis and service user status and described separately. Cost drivers were identified by means of a two-part regression model.

**Results:**

Total mean costs for 12 months for the total sample amount of € 3736.35 (95% CI: € 2816.84–4813.83) per person. Children with a psychiatric diagnosis generated a total of € 5691.93 (95% CI: € 4146.27–7451.38) of costs per person, compared to € 1245.01 (95% CI: € 657.44–1871.49) for children without a psychiatric diagnosis. The logit part indicates significant odds ratios for individual functioning and diagnosis of the child as well as for family functioning. The linear part reveals that increasing individual functioning in the child is related to decreasing costs.

**Conclusions:**

Children of families with parents with mental illness use a broad spectrum of mental health care, school-based support and youth welfare services even if they are not yet diagnosed as having a mental disorder. Further research should examine whether these institutions are sufficiently qualified and interlinked to meet the support needs of this vulnerable group.

*Trial registration* The study was registered at the 07/10/2014 before the start of data collection (04/11/2014) at the German clinical trials register (Deutsches Register Klinischer Studien, DRKS, nr: DRKS00006806, https://www.drks.de/drks_web/navigate.do?navigationId=trial.HTML&TRIAL_ID=DRKS00006806).

## Background

About three million children in Germany have at least one parent with mental illness (PMI) [1, 2]. Children concerned have a three to seven times higher lifetime risk of developing a mental illness themselves [3, 4]. When parents are mentally impaired, children have an increased psychosocial risk of experiencing socio-economic descent, interpersonal conflicts, separation of parents, or negligence [1], and mental health needs of children might not be recognised or get the necessary attention [5, 6]. This makes children of parents with mental illness (COPMI) more likely to develop mental health problems compared with children from the general population [4]. Therefore, measures to detect early signs of mental disorder in offspring of PMI and interventions to support families with PMI are recommended in the literature [7, 8]. However, research results regarding the effectiveness of such interventions are ambiguous: some interventions seem to be effective in terms of symptom reduction or decreased risk of diagnosis in children [9], while others did not find intervention effects on children’s mental health or social functioning [10]. Some detected medium to large effects on parents’ symptom severity and parenting behaviour [10], whereas no difference in the effects on children’s mental health could be found when comparing interventions for both parents and children, or interventions targeting parents only [9]. The approach of this study builds upon the recommendation of Bee et al. [10] to develop feasible and acceptable child- and family-based interventions. However, the cost-effectiveness of such interventions is rarely investigated and up to now, there had been only one in Germany [11].

As a basic requirement for health economic evaluations of health-related interventions, the whole spectrum of health-related costs incurred by the study participants must be estimated [12]. In case of interventions related to mental health this includes not only healthcare costs but also costs for psychosocial support, such as accommodation support and occupational rehabilitation [13]. Otherwise there is a risk of disregarding externalisation effects, caused by the shift of costs from the healthcare system to the social care system. For children and adolescents with mental health problems, comprehensive cost assessment must also consider costs of child welfare services and services provided by schools for behavioural problems [14].

Results from international studies indicate that COPMI use health as well as child and youth services more frequently than other children [15, 16]. In Germany, the use and the costs of health and social care services in COPMI are rarely investigated [10, 15]. In contrast to other countries (i.e. the UK), there is no comprehensive unit cost list for (children and adolescent mental) health and social care services in Germany. Based on a sample of the German population, Weschenfelder and colleagues (2018) recently estimated health costs for standard treatment of children with mental illness who are not attending school as being € 8020 for 12 months [11]. However, because healthcare costs cover only a part of the total societal costs of mental health problems in children and adolescents, this study provides insufficient basis for the health economic evaluation of preventive interventions.

This paper aims to: (a) investigate the whole spectrum of health and social services used by COPMI in Germany; (b) to provide a list of unit costs for these services and to estimate the corresponding costs for the society and the health system; and (c) to identify clinical and psychosocial characteristics which affect costs and service use.

## Methods

### Study design and participants

The sample of this investigation includes children and adolescents (CA) who participated in a randomised controlled trial on the evaluation of a prevention programme for families with at least one parent with mental illness (PMI, see study protocol [17]). Participating families were recruited at six study sites located at hospitals or hospital departments for adult or child and adolescent mental health in Germany between April 2014 and June 2017. Sites were selected on the basis of their particular interest in supporting families with PMI known from previous cooperations. Recruitment was carried out by means of posters, flyers, information during ward rounds, personal approach and patient-parent groups, as well as through newspaper advertisements. Families were included if they had at least one child aged between three and 19 years and if at least one parent reported having been diagnosed with a mental illness (F10 to F69 ICD-10) that was currently being treated or where treatment had finished recently. Diagnoses have been cross-checked with the patient records if available. Excluded from study participation were parents or children who experience severe psychopathological symptoms or suicidal thoughts indicating the need for acute inpatient care.

Because of the expected differences between the health and social care services used by COPMI and PMI, a comprehensive investigation of costs for both groups would go beyond the scope of this article. Therefore, the current paper focuses only on the costs of COPMI.

Diagnostic assessment of children was done by trained psychologists and psychotherapists. All other assessments were carried out by trained research workers on four separate occasions: before randomisation (t0), and at 6 (t1), 12 (t2), and 18 (t3) months follow-up. Health service use by all children was assessed with the help of their parents. The diagnostic interview, severity of mental illness, and functioning was assessed by asking parents about the health status of their child(ren) and also, for children aged ten years or older, by themselves. After providing informed consent, participants were randomly assigned to either the intervention group or the control group with treatment as usual.

### Instruments

Health service use and medication was measured with the Child and Adolescent Mental Health Service Receipt Inventory (CAMHSRI) [18]. The CAMHSRI questionnaire was adapted to the German psychiatric care system for CA. The CAMHSRI consists of eight parts: inpatient care, outpatient care, inpatient social services, outpatient social services, other inpatient services, school help, type of school, and medication. All types of services assess the number of consultations for the previous three months, except the parts about inpatient care, medication and type of school attended. Inpatient care and school type are assessed for the previous 12 months. The section about medication assesses the type, dosage and frequency of medication taken for the previous month. All services were assigned the corresponding cost per unit and afterwards extrapolated to 12 months. The CAMHSRI assessment took about ten minutes.

Diagnosis of children was assessed by semi-structured interviews with the German version of the Kiddie Schedule for Affective Disorders and Schizophrenia [19, 20]. Children below the age of ten have been assessed on the basis of their parents’ reports, while children from the age of ten years and up have been assessed directly. The German scale for assessing psychiatric disorders in CA (Skala zur Gesamtbeurteilung von Kindern und Jugendlichen — SGKJ) [21], the global assessment of functioning for adults (GAF) [22], the global assessment of relational functioning for adults (GARF) [23] and the clinical global impression score for adults (CGI) [24] were used for further analysis.

The SGKJ assesses current individual psychosocial functioning in CA on a hypothetical continuum and corresponds to the GAF for adults (Cronbach’s alpha 0.74 [25]), which also assesses current individual psychosocial functioning. The GARF assesses current relational functioning in adults. SGKJ, GAF and GARF are rated on a scale from 1 (dysfunctional) to 100 (fully functional). The SGKJ and the GAF distinguish in ten sections of ten points each, graduating individual functioning with higher values rating better functioning. The GARF is used as an observational instrument and addresses three major constructs (problem solving, organisation, and emotional climate) in five clinical vignettes (Cronbach’s alpha from 0.72 to 0.97 [26]). The CGI rates with a single item the severity of the mental illness of the adult patient at the time of inquiry. It is rated on the following seven-point scale: 1 = normal, not at all ill; 2 = borderline mentally ill; 3 = mildly ill; 4 = moderately ill; 5 = markedly ill; 6 = severely ill; 7 = among the most extremely ill patients. All scales enter analyses as raw scores.

### Costs and use of resources

As there is no unit cost list in Germany, costs for each service have been obtained from several sources. Information on inpatient costs has been taken from the German psychiatric system of diagnosis-related groups, called PEPP (Entgeltsystem Psychiatrie, Psychotherapie und Psychosomatik) [27], costs of office-based physicians have been calculated on the basis of the Doctors' Fee Schedule within the German Statutory Health Insurance Scheme (Einheitlicher Bewertungsmaßstab, EBM) [28]. Costs of services provided by the child welfare system have been acquired via telephone survey of authorities providing costs for child and youth social services (Table 2). Defined daily dose (DDD) prices for drugs and medication were determined based on active ingredient with the German report for pharmaceutical products [29].

### Statistical analyses

Standard errors and 95% confidence intervals for cost data have been estimated by means of nonparametrical bootstrapping with 1000 replications taking into account the clustering of children into families.

Regression based imputations have been performed to take into account missing values. Due to the high number of missing values, each of the imputed variables was imputed individually using costs, group and children’s diagnosis as explaining variables for the imputation. Imputed values were used for cost functions. Cost functions have been estimated by means of a two-part regression using a logistic model for the first part and a linear model with robust standard errors for the second part [30]. Both models took into account within-family clustering of the children [31]. Total annual costs were used as dependent variable (DV), age, gender, as well as baseline measures of children’s diagnosis, children’s SGKJ, parent’s mental health condition (diagnosis within the affective spectrum, CGI and GAF) and family functioning (GARF) as independent variables. A joint test was applied to confirm that all measures of parents are relevant for explaining the variance in total costs. Marginal effects (Delta-method) were calculated stepwise for all independent variables by stepwise addition of the variables into the two-part model. All analyses were performed using Stata 16.

## Results

In total, 215 families with 332 CA gave their consent to participate in the study. Parents were on average 42 years old, the participating parent was mostly the mother (N = 156, 75%) and about 52% (N = 111) of the parents reported diagnoses of affective disorders (ICD-10, F32 and F33).

On average, the participating CA were about 12 years old and 172 (52%) were female. Fifty-five percent (N = 186) were diagnosed as having a mental disorder at study baseline (Table 1). Details on services, frequency, and unit costs of service utilisation are presented in Table 2. The most-reported health-related services were: CA psychiatry (n = 28), CA psychiatrist (n = 44), psychotherapist (n = 54), paediatrician and general practitioner (GP, n = 62), and occupational therapist (n = 24). The most-used outpatient child and youth services were socio-pedagogical family assistance (n = 36) and parent–child counselling centres (n = 22). In the cases of 154 persons, no services were used. Details on different drug ingredients, DDD prices and total costs for each drug are displayed in Table 3 [29].

**Table 1:** Sample characteristics

Sample size children and adolescents (N)	332	Missing values
Age (m, SD)	11.7 (4.4)	3
Female gender (n, %)	172 (52)	3
With psychiatric diagnosis child (n, %)	186 (55)	–
SGKJ^a^ (m, SD, 0–100)	73.3 (13.4)	48
Psychiatric diagnosis of the parent within the depressive spectrum (n, %)	167 (50)	–
GARF^b^ (m, SD, 0–100)	62.4 (21.2)	41
CGI^c^ (m, SD, 0–10)	5.1 (1.1)	44
GAF^d^ (m, SD, 0–100)	55.9 (17.5)	51

^a^Global functioning of the child

^b^Global relational functioning of parents/family

^c^Clinical global impression for adults

^d^Global functioning for adults

**Table 2:** Unit costs of service utilisation

	Details	User (n)	Unit	Unit Costs (in €)	Source^a^	Year
Psychiatric services inpatient	Child and adolescent psychiatry	14	1 day	375.00	InEK GmbH [49]	2017
	Psychiatric department of a general hospital	2	1 day	375.00	InEK GmbH [49]	2017
	Paediatric clinic	2	1 day	375.00	InEK GmbH [49]	2017
	Parent–child-cure	3	1 day	92.00	Vdek [50]	2017
	Children’s protectory	1	1 day	172.20	Cooperative educational work [51]	2017
	Psychiatric rehabilitation for children	1	1 day	179.45	German federal pension fund [52]	2017
	Psychiatric day hospital	1	1 day	238.47	InEK GmbH [49]	2017
Psychiatric services outpatient	Child and adolescent psychiatrist	28	10 min	26.54	KBV [28]	2017
	Child and adolescent psychotherapist	28	50 min	88.56	KBV [28]	2017
	Paediatrician	33	1 visit	20.32	KBV [28]	2017
	GP	14	1 visit	20.32	KBV [28]	2017
Other outpatient	Obesity intervention programme	1	90 min	85.53	City of Leipzig [53]	2018
	Occupational therapist	15	45 min	34.82	KVBB [54]	2017
	Physiotherapist	2	30 min	27.46	Buchner [55]	2011
	Neurologist	1	1 visit	24.64	KBV [28]	2017
	Osteopath	1	40 min	105.00	Osteopaths association [56]	2017
	Homeopath	1	40 min	60.00	NAV-Virchow-association [57]	2013
	Alternative practitioner	2	30 min	12.30	Association of alternative practitioners [58]	2002
	Remedial teacher	2	60 min	50.87	Herzog [59]	2018
	Speech therapist	8	45 min	35.91	KVBB [60]	2017
	Orthodontist	1	1 visit	14.06	BZAEK [61]	2011
	Endocrinologist	1	1 visit	17.48	KBV [28]	2017
	Diabetologist	1	1 visit	17.48	KBV [28]	2017
	Socio-paediatric centre	1	3 months	344.49	KJA-SPZ Berlin [62]	2018
	Art therapy	1	90 min	100.00	Holzmann [63]	2018
	Acupuncture	1	1 visit	11.66	Medical fee schedule [64]	2020
Child and youth services inpatient	Assisted living for adolescents	1	1 day	141.10	Cooperative educational work [51] and Child protective services Leipzig [65]	2017
	Assisted living for children	1	1 day	110.00	Child protective services Leipzig [65]	2018
	Foster family (long-term)	2	1 day	30.51	Child protective services Günzburg [66]	2018
	Foster family (short-term)	2	1 day	57.50	Child protective services Günzburg [66]	2018
	Parent–child facility	1	1 day	75.00	Administrative district office Neu-Ulm [67]	2018
Child and youth services outpatient	Socio-paedagogical family assistance	29	60 min	38.50	County council Pinneberg [68]	2010
	Parent–child counselling centre	13	60 min	38.50	County council Pinneberg [68]	2010
	Socio-paedagogical day-care	3	1 day	133.70	Klein-Jung [69]	2017
	Child and adolescent emergency service	2	10 min	20.32	KBV [28]	2017
	Nutrition counselling	1	60 min	80.00	Ziegert [70] and Conze [71]	2018 and 2018
	Church counselling centre	2	60 min	38.50	County council Pinneberg [68]	2010
School help	Classroom teacher	35	45 min	23.61	Holzapfel [72]	2018
	Social worker	9	60 min	38.50	County council Pinneberg [68]	2010
	Educational psychologist	6	50 min	88.56	KBV [28]	2017
	School companion	1	45 min	27.00	Administrative District Office Günzburg [73]	2018
Kind of School	Special needs school	15	1 day	51.45	State Ministry of Education of Saxony [74]	2016
	Speech therapy school	2	1 day	51.45	State Ministry of Education of Saxony [74]	2016

^a^Glossary: InEK = National Institute for Hospital Reimbursement (Institut für Entgeltsysteme im Krankenhaus); vdek = Association of Health Insurance Companies; KBV = National Association of Statutory Health Insurance Physicians; KVBB = Brandenburg Association of Statutory Health Insurance Physicians; BZAEK = German Federal Association of Dentists; KJA-SPZ = coordination centre of socio-paediatric care Berlin

**Table 3:** Overview about costs of taken drugs [29]

Active ingredient	Duration of intake (days)	DDD costs (in €)	Total costs (in €)
Bupropion	30	1.05	31.50
Citalopram	30	0.16	4.80
Escitalopram	31	1.23	38.13
Fluoxetine	14	0.23	3.22
Lamotrigine	30	0.84	25.20
Lisdexamfetamine	30	3.10	93.00
Methylphenidate	30	1.24	37.20
Pramipexole	30	5.47	164.10
Prothipendyl	30	1.28	38.40
Quetiapine	30	6.41	192.30
Valproic acid	30	0.92	27.60
Zopiclon	14	0.67	9.38

*DDD* Defined Daily Dose, N = 297 (94%) did not take any drugs

**Fig. 1:**
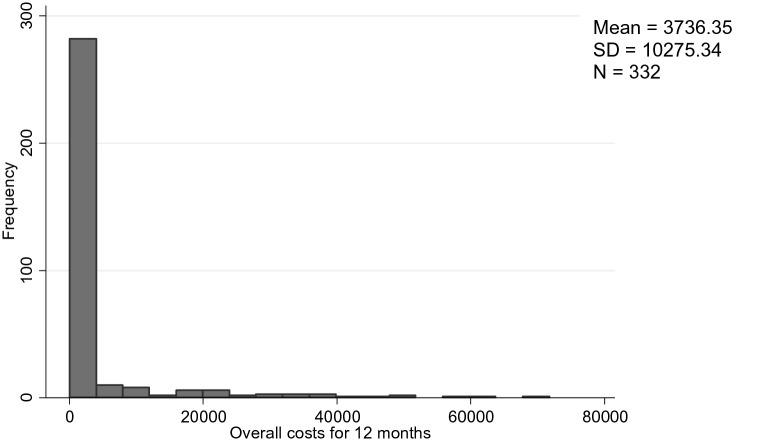
Total cost for 12 months

The total mean costs for 12 months for the total sample amount to € 3736.35 (95% CI: € 2816.84–4813.83) per person. CA with diagnosis generated a total of € 5691.93 (95% CI: € 4146.27–7451.38) of costs per person, compared to € 1245.01 (95% CI: € 657.44–1871.49) for children without psychiatric diagnosis (Table 4). Figure 1 shows that the distribution of total costs is positively skewed, common for healthcare cost data (see Fig. 1). Mean inpatient costs amount to € 1549.70 (95% CI: € 897.57–2369.93), outpatient costs to € 383.20 (95% CI: € 283.52–489.67), inpatient child and youth services to € 442.13 (95% CI: € 133.44–809.13), outpatient child and youth services to € 258.25 (95% CI: € 170.53–355.90), school services to € 1063.89 (95% CI: € 641.75–1576.14) and medication to € 39.19 (95% CI: € 15.96–70.67). Children with diagnosis generated significantly higher costs in psychiatric inpatient (p = 0.007) and outpatient services (p < 0.001), as well as in inpatient youth services (p = 0.053), medication (p = 0.043) and total costs (p < 0.001), compared to children without psychiatric diagnosis.

**Table 4:** Total costs and 95% confidence intervals (CI) for 12 months

		Total sample			Comparison With / Without Diagnosis	Children who generated any service related costs			Comparison With/Without Diagnosis
		Total N = 332 (100%)	With Diagnosis N = 186 (56%)	No Diagnosis N = 146 (44%)	F-test, bootstrapped (p-value)	Total N = 144*** (100%)	With Diagnosis N = 107 (74%)	No Diagnosis N = 37 (26%)	F-test, bootstrapped (p-value)
Psychiatric services inpatient	Mean 95% CI*	1549.70 (897.57–2369.93)	2540.40 (1350.75–4003.36)	287.57 (35.17–703.71)	*0.007*	3572.91 (1962.38–5687.61)	4416.02 (2385.32–7047.48)	1134.73 (126.97–2806.96)	0.123
Psychiatric services outpatient	Mean 95% CI	383.20 (283.52–489.67)	581.38 (414.83–760.57)	130.73 (57.41–210.02)	* < 0.001*	883.49 (642.82–1138.15)	1010.61 (740.74–1325.56)	515.85 (252.26–815.64)	0.099
Child and youth services inpatient	Mean 95% CI	442.13 (133.44–809.13)	789.19 (249.51–1476.20)	0 (0)	0.053	1019.37 (276.74–2157.76)	1371.86 (407.19–2605.06)	0 (0)	0.198
Child and youth services outpatient	Mean 95% CI	258.25 (170.53–355.90)	326.89 (176.50–497.82)	170.81 (77.43–266.40)	0.180	595.42 (380.66–879.80)	568.24 (313.94–843.81)	674,00 (327,33–1053,00)	0.719
School help	Mean 95% CI	1063.89 (641.75–1576.14)	1384.13 (805.45–2033.65)	655.90 (159.46–1137.87)	0.159	2285.72 (1324.25–3444.69)	2181.14 (1166.45–3317.61)	2588.15 (806.73–4463.19)	0.748
Medication	Mean 95% CI	39.19 (15.96–70.67)	69.95 (24.84–126.85)	0 (0)	0.043	90.35 (34.02–194.26)	12.59 (42.62–222.30)	0 (0)	0.177
Overall	Mean 95% CI	3736.35 (2816.84–4813.83)	5691.93 (4146.27–7451.38)	1245.01 (657.44–1871.49)	* < 0.001*	8447.25 (6392.95–10,840.61)	9669.46 (6994.37–12,659.32)	4912.73 (2722.11–7105.98)	0.078

*Nonparametric, bias corrected bootstrapping with 1000 replications taking into account within-family clustering, **Percentiles; ***No costs N = 188

Six variables were imputed with the number of imputed observations in brackets: age (3), gender (3), SGKJ (48), GAF (40), CGI (32) and GARF (29). The logit part of the two-part regression model indicates significant odds ratios (OR) for individual functioning and diagnosis of the child as well as for family functioning (GARF, see Table 5, first part). The linear part of the two-part regression model reveals that increasing the age of the child is related to increasing costs (b_age_ = 618.51; p = 0.037) while increasing individual functioning in the child is related to decreasing costs (b_SGKJ_ = -368.39; p = 0.002). The logit part of the model explains about 17% of the probability for using any type of service, whereas the linear part explains about 23% of total cost variance for all cases with costs > 0. A joint test of the common effect of parental functioning (GARF, GAF and CGI) showed a chi^2^(6) value of 14.96 (p = 0.021). Average marginal effects for age and functioning of the child differ only slightly between the linear model and the linear part of the two-part regression model and between the imputed and the not-imputed models. Type A error levels did not differ with regard to the 5% significance criterion. The average marginal effect of age amounts to a 325.42 Euro increase in costs per year of increased age. With each one-unit increase in the SGKJ (functioning of the child), there is a 213.94 Euro decrease in costs (Table 6, the marginal effects of the linear regression model and the not-imputed models can be found in Additional file 1).

**Table 5:** Model 1–Imputed two-part regression model for all participants with robust estimates

Model	Odds ratio	p-value	95% confidence interval for B	
			Lower bound	Upper bound
*Part 1: Logit N* = *332, Prob* > *chi*^*2*^ = *0.0000, Pseudo-R*^*2*^ = *0.1726*				
(constant)	0.800	0.625	− 2.406	4.006
Age (child)	0.043	0.161	− 0.017	0.102
Gender (child, male = 0, female = 1)	− 0.017	0.951	− 0.551	0.518
Diagnosis of the child	0.933	0.003	0.323	1.543
SGKJ^a^ child	− 0.040	0.005	− 0.068	− 0.012
Parental diagnosis (depressive spectrum = 1)	0.288	0.335	− 0.297	0.874
GARF^b^ of PMI	− 0.025	0.002	− 0.041	− 0.009
CGI^c^ of PMI	0.255	0.121	− 0.067	0.578
GAF^d^ of PMI	0.016	0.065	− 0.001	0.033

	Regression coefficient B	p-value	95% confidence interval for B	
			Lower bound	Upper bound
*Part 2: Regress N* = *145, Prob* > *F* = *0.0001, R*^*2*^ = *0.2308*				
(constant)	38,935.72	0.001	15,710.45	62,160.99
Age (child)	618.506	0.037	37.255	1199.758
Gender (child, male = 0, female = 1)	− 450.433	0.844	− 4932.417	4031.551
Diagnosis of the child	− 585.133	0.790	− 4899.812	3729.546
SGKJ^a^ child	− 368.392	0.002	− 607.042	− 129.741
Parental diagnosis	− 1369.723	0.552	− 5881.684	3142.237
GARF^b^ of PMI	108.418	0.137	− 34.355	251.190
CGI^c^ of PMI	− 786.296	0.496	− 3047.756	1475.164
GAF^d^ of PMI	− 232.240	0.073	− 486.050	21.571

Dependent variable: total costs for 12 months

^a^Global functioning of the child

^b^Global relational functioning of parents/family

^c^Clinical global impression

^d^Global functioning of the PMI

Part 1: replications based on 213 clusters (families)

Part 2: replications based on 109 clusters (families)

**Table 6:** Marginal effects of the imputed two-part model

N = 332	Two-part model		
	dy/dx	Standard error (SE)	p-value*
Age (imp.)	325.42	139.95	*0.020*
Gender (imp.)	− 219.04	1062.33	0.837
Diagnosis (child)	1094.00	1032.64	0.289
SGKJ (imp.)	− 213.94	55.83	*0.000*
Diagnosis (parent)	− 217.47	1076.19	0.840
GARF (imp.)	14.14	32.04	0.659
CGI (imp.)	− 4.76	530.41	0.993
GAF (imp.)	− 80.12	56.57	0.157

* Significant p < 0.05

## Discussion

To our knowledge, this is the first study investigating the use and the costs of health and psychosocial services used by COPMI in Germany.

Our results reveal that 43% of the participating CA reported having used at least one health or social service unit. As indicated by the comparison of service categories, about 50% of the total costs were incurred by psychiatric inpatient services while about 30% were incurred by non-medical services provided by child and youth welfare authorities and schools. Although costs for all service categories were significantly higher for participants diagnosed as having a mental illness, one third of the participants without a current diagnosis reported the use of at least one service unit including psychiatric inpatient treatment. These results underline that comprehensive estimation of costs associated with having a PMI should include the whole spectrum of services provided for emotional and behavioural problems in CA. Furthermore, the fact that the use of treatment and support is not limited to those CA who have been diagnosed as having a current mental disorder may either indicate that the diagnostic procedure applied in our study was not sensitive enough to identify all cases with a mental disorder, or that there is a substantial need for services below the threshold of a diagnosis in our target group. Especially the subgroup of children without diagnosis but using services (26%, N = 37) generating mean costs of € 1134.73 in the psychiatric inpatient sector, suggests that these children are not diagnosed correctly or not treated adequately or in the adequate system. To answer the question of to what extent these explanations are appropriate, representative samples of families with PMI would be needed.

The difference in results between children with and without diagnosis can be explained by the fact that even if behavioural problems already occurred in educational or welfare settings, mental disorders are in most cases only diagnosed for the first time by psychologists or psychiatrists in mental health care facilities. Accordingly, the fact that school-based support services are most widely used by children without mental health diagnosis indicates that staff providing these services may detect behavioural problems at a lower threshold [32]. Results of our cost regression model reveal that the probability of using any service is associated with the mental health-related characteristics of the children as well as those of the PMI, while the intensity and the costs of service use is associated with the age and functional capacity of the child.

These results may reflect the fact that parents’ knowledge and appraisal of mental health problems of their children determine their help-seeking behaviour [33, 34]. This implies parents’ awareness of their children’s mental health care needs, but in case of the presence of the parents’ own mental illness, this awareness might be lacking, resulting in non or delayed help-seeking [33]. CA have a mean delay in help-seeking of about four years [35]. Due to the lack of awareness, this time might be even longer in case of COMPI, resulting in an externalisation of healthcare costs to the educational or the child welfare system. In addition to lacking awareness of CA mental health needs, PMI might delay in help-seeking for their children due to shame or fear of stigmatisation [36, 37], lacking mental health literacy [38], or due to their reluctance to reveal their own mental illness to their children, teachers or educational staff [39]. However, the results may also reflect the fact that, in Germany, support for mental health problems in children or adolescents is usually provided by different facilities and also differently financed than support for adults with mental health problems. As a consequence, services for the support of families with PMIs rarely exist and the problems resulting from parenthood and mental disorder are only considered by the health and social care system if they become obvious due to significant behavioural problems of the children or adolescents.

Literature indicates that there is a relationship between caregivers’ mental health and caregiving skills [40], as well as between caregivers’ mental health and low social support [41]. Caregiving skills were not measured in this trial but we found a significant effect upon family functioning, indicating that an increased level of functioning is related to a higher probability of using any type of health or social service and of incurring costs in the first part of the model. Family functioning is known to be correlated with the mental health of COPMI [42], which is also true for our sample (r = 0.344, p < 0.001), indicating a good resilience in participating children. Further investigations about resilience in COPMI and the influence of individual and family functioning on costs are needed. Still, preventive interventions targeting family functioning are shown to be effective [43] and might be cost-saving in the long run.

In our sample, 56% of COPMI have a psychiatric diagnosis themselves. This is consistent with previous findings in the literature. Mattejat et al. [44] for example showed that about 50% of children and adolescents showing up in mental health services live with a PMI. Campbell et al. [45] even report a prevalence of mental illness of up to 79% in parents of children receiving mental health treatment. Van Santvoort et al. [46] confirmed in their review the message of Cicchetti et al. [47, 48] that COPMI are at risk of developing mental illness—either the same as their parents or another disorder—with a strong tendency for the same disorder as their parents.

Special attention is needed for children who do have a diagnosis but who reported no costs (N = 79), indicating a lack of treatment. Therefore, there is a need to offer early help for the children of PMI as well as to raise awareness in other family members, caregivers, or GPs for noticeably different functioning and behaviour in the child.

### Strengths and limitations

This study is the first study presenting primary data on comprehensive health and social care service use and costs of COPMI in Germany. This paper presents a unit cost list for health and social care services for CA with mental health problems in the German healthcare system, therefore adding significant information about youth and social service costs to recently published healthcare costs [11]. In contrast to previous studies we included the full range of school-based and child welfare services.

Limitations of the study need to be considered. First, since the participating families have been recruited in mental health service facilities, the study sample is not representative for COPMI, which limits the generalisability of our results. Second, participating parents or children might not recall all used services or drugs which can possibly lead to an underestimation of real costs. Third, assessment of the children’s psychological status via the report of parents may furthermore result in an underreporting of psychological problems in children below the age of ten. Fourth, the influence of parental diagnoses apart from depression might be underestimated, as other diagnoses are less frequent in the spectrum of mental disorders. Fifth, the use of simple regression-based imputation may underestimate the variance of the imputed variables. Sixth, since we did not measure service needs directly we can estimate the proportion of unmet service needs only indirectly. Seventh, the extrapolation of service use to 12 months might overestimate the frequency of service use and the average costs among those who use services, while the proportion of persons with any service use might be underestimated.

## Conclusions

While our results in general reveal that mental and social care services are provided to those children who need support, we also identified 79 children (24%) with a diagnosis of a mental disorder who did not report any use of mental or social care services. This indicates that a significant proportion of COPMI might be disregarded by the current system of mental and social care. On the other hand, the fact that 37 of the children in our sample (11%) reported the use of mental or social care services indicates that the need for support may already exist below the threshold of a clinical diagnosis. Given the fact that the risk of being disregarded by the mental and social care system is higher for those without a diagnosis than for those who have already been diagnosed, we would expect that the proportion of children with unmet needs for support might be considerable.

## Supplementary Information

**Additional file 1.** Additional tables.

## Data Availability

Participants provided written informed consent under the condition of confidentiality of their data including restricted access of third parties. Therefore data cannot be shared.

## Abbreviations

CA: Children and adolescents
CAMHSRI: Child and Adolescent Mental Health Service Receipt Inventory
CGI: The clinical global impression score (in parents)
COPMI: Children of parents with mental illness
GAF: The global assessment of functioning (in parents)
GARF: The global assessment of relational functioning (in parents)
GP: General Practitioner
ICD: International Statistical Classification of Diseases and Related Health Problems
OR: Odds ratio
PMI: Parent with mental illness
SGKJ: The German scale for assessing psychiatric disorders in children and adolescents
