# Supplementary material for: Costs of health and social services use in children of parents with mental illness
Source: Child Adolesc Psychiatry Ment Health. 2021 Feb 20;15:10. doi: 10.1186/s13034-021-00360-y (PMC7897390; doi:10.1186/s13034-021-00360-y)
Supplement: Supplementary file 1 — Additional file 1. Additional tables. [file 13034_2021_360_MOESM1_ESM.docx]

Additional file for

**Costs of health and social services use in children of parents with mental illness**

Tamara Waldmann^1*^, Maja Stiawa^1^, Ümügülsüm Dinc^1^, Gülsah Saglam^1^,
Mareike Busmann², Anne Daubmann^3^, Bonnie Filter², Karl Wegscheider^3^,
Silke Wiegand-Grefe², Reinhold Kilian^1^

^1^ Department of Psychiatry and Psychotherapy II, University of Ulm and BKH Günzburg, Germany

² Department of Child and Adolescent Psychiatry and Psychotherapy, University Medical Center Hamburg-Eppendorf, Germany

^3^ Department of Medical Biometry and Epidemiology, University Medical Center Hamburg-Eppendorf, Germany

Table S1 Results of the imputed linear regression model (N=332)

| model | Observed coefficent | Boot strapped  Standard Error | p-value | 95.0% confidence interval for B | |
| --- | --- | --- | --- | --- | --- |
| *Prob>chi² = 0.0001, R² = 0.198  R²adjusted = 0.178* |  |  |  | Lower Bound | Upper Bound |
| (constant) | 21755.45 | 7015.92 | .002 | 8004.50 | 35506.4 |
| **age (child)** | 372.27 | 124.39 | **.003** | 128.47 | 616.08 |
| gender  (child, male = 0, female =1) | -117.56 | 1030.94 | .909 | -2138.16 | 1903.05 |
| diagnosis of the child | 11.35 | 984.98 | .991 | -1919.18 | 1941.88 |
| **SGKJ^a^ child** | -306.01 | 69.93 | **.000** | -443.08 | 168.95 |
| parental diagnosis  (depressive spectrum = 1) | -288.55 | 1127.33 | .798 | -2498.06 | 1920.97 |
| GARF^b^ of PMI | 21.70 | 32.68 | .507 | -42.35 | 85.74 |
| CGI^c^ of PMI | 566.74 | 545.70 | .299 | -502.81 | 1636.30 |
| GAF^d^ of PMI | -65.08 | 52.94 | .219 | -168.83 | 38.68 |

*bootstrapped with 1000 replications taking into account within-family clustering

Table S2 Results of the not-imputed linear regression model (N = 256)

| model | Observed coefficent | Boot-strapped  Standard Error | p-value | 95.0% confidence interval for B | |
| --- | --- | --- | --- | --- | --- |
| *Prob>chi² = 0.0006, R² = 0.210  R²adjusted = 0.184* |  |  |  | Lower Bound | Upper Bound |
| (constant) | 26467.8 | 8570.16 | .882 | 9670.60 | 43265 |
| **age (child)** | 363.75 | 146.96 | **.013** | 75.71 | 651.79 |
| gender  (child, male = 0, female =1) | -476.16 | 1272.69 | .708 | 2970.58 | 2018.26 |
| diagnosis of the child | -433.07 | 1217.71 | .722 | 2819.74 | 1953.59 |
| **SGKJ^a^ child** | -347.35 | 81.73 | **.000** | 507.53 | 187.17 |
| parental diagnosis  (depressive spectrum = 1) | -210.82 | 1424.89 | .882 | 3003.54 | 2581.91 |
| GARF^b^ of PMI | 32.33 | 48.56 | .506 | -62.84 | 127.51 |
| CGI^c^ of PMI | 414.61 | 652.57 | .525 | -864.40 | 1693.61 |
| GAF^d^ of PMI | -80.74 | 71.93 | .262 | -221.73 | 60.25 |

*bootstrapped with 1000 replications taking into account within-family clustering

Table S3 Results of the not-imputed two-part model (N = 256)

| model | Odds ratio | p-value | | 95.0% confidence interval for B | | | |
| --- | --- | --- | --- | --- | --- | --- | --- |
|  |  |  |  | Lower Bound | | Upper Bound | |
| ***Part 1:*** *Logit N=332, Prob>chi² = 0.0000, Pseudo-R² = 0.1726* |  |  |  | |  | |  |
| (constant) | 1.18 | .486 | -2.14 | | .03 | |  |
| age (child) | .04 | .257 | -.03 | | .10 | |  |
| gender  (child, male = 0, female =1) | -.14 | .648 | -.76 | | .47 | |  |
| diagnosis of the child | .65 | .052 | -.01 | | 1.30 | |  |
| **SGKJ^a^ child** | -.04 | **.010** | -.07 | | -.01 | |  |
| parental diagnosis  (depressive spectrum = 1) | .28 | .408 | -.39 | | .95 | |  |
| **GARF^b^ of PMI** | -.03 | **.007** | -.05 | | -.01 | |  |
| CGI^c^ of PMI | .29 | .130 | -.08 | | -.01 | |  |
| GAF^d^ of PMI | .01 | .151 | -.01 | | .65 | |  |
|  |  |  |  | |  | |  |
|  | regression coefficient B | p-value | 95.0% confidence interval for B | | | |  |
|  |  |  | Lower Bound | | Upper Bound | |  |
| ***Part 2:*** *Regress N= 145, Prob > F = 0.0001, R² = 0.2308* |  |  |  | |  | |  |
| (constant) | 47491.02 | .000 | 22268.2 | | 72713.84 | |  |
| age (child) | 553.78 | .090 | -86.28 | | 1193.84 | |  |
| gender  (child, male = 0, female =1) | -611.71 | .821 | -5910.51 | | 4687.08 | |  |
| diagnosis of the child | -678.98 | .801 | -5959.81 | | 4601.85 | |  |
| **SGKJ^a^ child** | -462.81 | **.001** | -725.62 | | -200.01 | |  |
| parental diagnosis | -955.38 | .716 | -6110.28 | | 4199.53 | |  |
| GARF^b^ of PMI | 175.51 | .102 | -34.91 | | 385.94 | |  |
| CGI^c^ of PMI | -1288.18 | .358 | -4035.78 | | 1459.42 | |  |
| GAF^d^ of PMI | -283.31 | .064 | -583.26 | | 16.63 | |  |

Part 1: replications based on 168 clusters (families)

Part 2: replications based on 88 clusters (families)

Table S4 Average marginal effects of the imputed and not-imputed linear regression model and the two-part models

|  | imputed models N = 332 | | | | | | not-imputed models N = 256 | | | | | | |
| --- | --- | --- | --- | --- | --- | --- | --- | --- | --- | --- | --- | --- | --- |
|  | Linear regression | | | Two-part model | | | Linear regression | | | Two-part model | | | |
|  | dy/dx | standard error (SE) | p-value* | dy/dx | standard error (SE) | p-value | dy/dx | standard error (SE) | p-value | dy/dx | standard error (SE) | p-value | |
| **Age**  **(imp.)**** | 371.40 | 126.56 | **.004** | 325.42 | 139.95 | **.020** | 363.75 | 146.99 | **.014** | 311.25 | 162.55 | .056 | |
| Gender (imp.) | -159.18 | 998.92 | .874 | -219.04 | 1062.33 | .837 | -476.16 | 1269.30 | .706 | -488.53 | 1322.06 | .712 | |
| Diagnosis (child) | 5.41 | 1024.10 | .996 | 1094.00 | 1032.64 | .289 | -433.07 | 1280.75 | .721 | 686.37 | 1319.02 | .603 | |
| **SGKJ (imp.)** | -306.18 | 70.64 | **.000** | -213.94 | 55.83 | **.000** | -347.35 | 77.93 | **.000** | -269.68 | 65.89 | **.000** | |
| Diagnosis (parent) | -287.25 | 1112.36 | .800 | -217.47 | 1076.19 | .840 | -210.82 | 1398.29 | .880 | -48.93 | 1310.79 | .970 | |
| GARF (imp.) | 21.80 | 32.03 | .497 | 14.14 | 32.04 | .659 | 32.33 | 45.88 | .482 | 44.34 | 49.70 | .372 | |
| CGI  (imp.) | 567.16 | 506.44 | .264 | -4.76 | 530.41 | .993 | 414.61 | 604.42 | .494 | -192.42 | 680.46 | .777 |  |
| GAF (imp.) | -65.20 | 51.98 | .211 | -80.12 | 56.57 | .157 | -80.74 | 65.77 | .221 | -111.47 | 71.51 | .119 |  |

*p<0.05 **if variable imputed, named in brackets
